# A combined experimental and computational framework to evaluate the behavior of therapeutic cells for peripheral nerve regeneration

**DOI:** 10.1002/bit.28105

**Published:** 2022-05-02

**Authors:** Despoina Eleftheriadou, Maxime Berg, James B. Phillips, Rebecca J. Shipley

**Affiliations:** ^1^ Centre for Nerve Engineering University College London London UK; ^2^ Department of Pharmacology, UCL School of Pharmacy University College London London UK; ^3^ Department of Mechanical Engineering University College London London UK

**Keywords:** glucose, hypoxia, mathematical modeling, microenvironment, tissue engineering, VEGF

## Abstract

Recent studies have explored the potential of tissue‐mimetic scaffolds in encouraging nerve regeneration. One of the major determinants of the regenerative success of cellular nerve repair constructs (NRCs) is the local microenvironment, particularly native low oxygen conditions which can affect implanted cell survival and functional performance. In vivo, cells reside in a range of environmental conditions due to the spatial gradients of nutrient concentrations that are established. Here we evaluate in vitro the differences in cellular behavior that such conditions induce, including key biological features such as oxygen metabolism, glucose consumption, cell death, and vascular endothelial growth factor secretion. Experimental measurements are used to devise and parameterize a mathematical model that describes the behavior of the cells. The proposed model effectively describes the interactions between cells and their microenvironment and could in the future be extended, allowing researchers to compare the behavior of different therapeutic cells. Such a combinatorial approach could be used to accelerate the clinical translation of NRCs by identifying which critical design features should be optimized when fabricating engineered nerve repair conduits.

## Graphical abstract

Combining in vitro experiments and mathematical modeling to quantify the behavior of therapeutic cells in peripheral nerve injury repair scenarios.

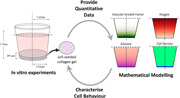

## 1 INTRODUCTION

Peripheral nerve injuries (PNIs) are associated with high socioeconomic and personal costs; the mean patient age is ~30 years so they can greatly impact lifetime health and productivity (Lad et al., 2010). Although nerves exhibit some regenerative capacity, the degree of reinnervation and subsequent recovery is dependent on many factors. In the case of incomplete regeneration, patients might not achieve meaningful functional recovery and the resulting disability may be highly debilitating with long‐term effects on them and their families (Grinsell & Keating, 2014; Panagopoulos et al., 2017).

The current “gold standard” of treatment for large gap PNIs is an autograft, where a sensory nerve is harvested and sutured to bridge the gap. However, autografts exhibit success rates that are far from ideal (Yang et al., 2011), as well as causing donor site morbidity and being limited in their availability. Recently, research in PNI treatment has focused on nerve repair constructs (NRCs), that combine therapeutic cells and biomaterials. When implanted in the injury site, NRCs can provide mechanical support, guidance cues and a growth‐permissive environment to modulate regeneration (Carriel et al., 2014; Hsu et al., 2013; Schuh et al., 2018). Research into the design of NRCs has so far focused predominantly on the choice of biomaterial, cell type and proregenerative cues. For NRCs that include a cellular component, key aspects that require optimization include spatial distribution of embedded cells and long‐term nutrient supply.

The conditions in the local microenvironment, particularly the native low oxygen levels, are a major determinant of the regenerative success of cellular NRCs that are often overlooked. Under physiological conditions the characteristic penetration length of oxygen in tissue is considered to be around 100–200 μm, depending on the proximity to blood vessels, cell type, and density (Carrier et al., 2002; Rouwkema et al., 2008). PNIs cause acute damage and disruption to microvascular networks thereby obstructing tissue perfusion (Lim et al., 2015). The resulting local tissue hypoxia and absence of neovascularisation can affect oxygen diffusion and distribution within implanted NRCs. As a result, the core of the NRC, which often lies at a distance beyond the diffusion distance of oxygen away from the nerve stumps, may become severely hypoxic compared to the ends of the NRC. Besides the physical characteristics of the biomaterial used, oxygen consumption rates are also determined by the type of cells embedded in the construct (Cheema et al., 2012; Magliaro et al., 2019; McMurtrey, 2015). For instance, pluripotent stem cells tend to have low metabolic rates, while progenitor and differentiated cells have higher metabolic rates (Teslaa & Teitell, 2015). Additionally, the consumption rate of three‐dimensional (3D) cultures appears to change based on the cell seeding density (Magliaro et al., 2019; Patzer II, 2004; Sielaff et al., 1997).

The formation of oxygen gradients has been found to correlate with gradients in viable cell density (Lewis et al., 2005; Radisic et al., 2006) and increased metabolic loads of the embedded cells (Carrier et al., 2002). Besides cell viability, oxygen availability is also linked to vascular regeneration. Cells in hypoxic environments often respond by the activation of multiple proangiogenic pathways and the upregulation of factors that encourage new vessel formation (Fong, 2008; Hashimoto & Shibasaki, 2015). Therefore, the presence of oxygen gradients within an NRC can lead to the formation of growth factor gradients, such as vascular endothelial growth factor (VEGF), which in turn can result in a more distinct directional chemotactic response of migrating endothelial cells. This can have further implications for PNIs as VEGF expression and neovascularisation have been found to induce axonal regrowth and Schwann cell proliferation and promote neural regeneration (Cattin et al., 2015; Donzelli et al., 2016; Hobson et al., 2000).

Improving our understanding of the impact of the local microenvironment on implanted cells is therefore beneficial for informing NRC design. Nevertheless, most in vitro studies assessing the behavior and proregenerative capacity of NRCs, have been performed at standard laboratory incubator oxygen concentrations. This condition does not represent the local in vivo endoneurial oxygen tension that studies in rat sciatic and human sural nerves report to be around 3%–7% (Newrick et al., 1986; Tuck et al., 1984; Zochodne et al., 1994). For this purpose, here, the effect of in vitro oxygen conditions on cell survival, VEGF release, as well as oxygen and glucose consumption, were measured. To simulate the sorts of cellular biomaterial used in translational nerve tissue engineering research, differentiated neural stem cells (CTX0E03) at a range of cell densities were embedded in thin, stabilized collagen constructs, and subsequently cultured at a physiologically relevant range of oxygen concentrations as well as standard cell culture conditions. CTX0E03 cells were selected as they are human clinical‐grade therapeutic cells with demonstrated potential as an allogeneic “off the shelf” cell source for peripheral nerve repair (Kalladka et al., 2016; O'rourke et al., 2018; Rayner et al., 2021; Smith et al., 2012).

The in vitro cellular biomaterial model allows us to explore NRC performance in a highly controlled environment, providing an insight into the behavior of therapeutic cells in the critical first 24 h after implantation. However, while in vitro experiments are invaluable in furthering our understanding of cellular behavior and in improving NRC design, the vast number of possible scenarios, including differences in cell density and distribution, biomaterial permeability, and anisotropy, that need to be tested can be prohibitive. To accelerate the design process, mathematical modeling can be integrated into experimental work to create an efficient and robust multidisciplinary workflow and allow for continuous improvement through an iterative process (Coy et al., 2018). Such tools can also be used to extrapolate data from in vitro studies to an in vivo repair environment, thus refining future in vivo studies.

To this end, we derived a cell‐solute model, which comprises a set of coupled partial differential equations describing the spatial and the temporal evolution of the CTX0E03 population and its local environment, including oxygen and glucose consumption and VEGF release. This allows us to assess the spatial gradients that will be established in repaired nerves and exploit this information in construct design (Coy et al., 2020, 2021). This type of mathematical model has been widely used to enhance the design of engineered tissues and tissue culture bioreactors (Cochran et al., 2006; McMurtrey, 2015; Rutkowski & Heath, 2002), as they are computationally cost‐effective, rely on a limited set of parameters, while still capturing most of the underlying biophysics. To calibrate this model, we first performed a sensitivity analysis of its outputs that allowed us to highlight the hierarchy between the different parameters. We then used the experimental observations to assign representative values to these parameters by matching the predictions of the mathematical model against the obtained data.

## 2 METHODS

Unless otherwise stated all cell culture materials were purchased from Sigma Aldrich or Thermo Fisher Scientific.

### 2.1 Culture and differentiation of CTX0E03 cells

Human neural stem cells (CTX0E03, level P28‐P31, ReNeuron Ltd) were cultured in Dulbecco's modified Eagle's medium:F12 medium supplemented with human albumin (0.03%; Nova Biologics); Glutamax (2 mM); human transferrin (5 μg/ml), putrescine dihydrochloride (16.2 μg/ml), human insulin (5 μg/ml; Sigma), progesterone (60 ng/ml; Sigma), sodium selenite (40 ng/ml), epidermal growth factor (20 ng/ml), basic fibroblast growth factor (10 ng/ml; Invitrogen), and 4‐hydroxytamoxifen 4‐OHT (100 nM) in 175 cm^2^ laminin‐coated (10 µg/ml; Amsbio) flasks. Following expansion, CTX0E03 cells were subsequently differentiated for 1 week by removal of growth factors and 4‐OHT.

### 2.2 Fabrication of stabilized cellular collagen gels

Differentiated CTX0E03 cells (dCTX0E03) were used to create stabilized cellular collagen scaffolds. These were used to mimic the conditions in engineered neural tissue constructs. All gels were prepared using 80% v/v type I rat tail collagen (2 mg/ml in 0.6% acetic acid; First Link) mixed with 10% v/v 10× minimum essential medium. The mixture was then neutralized using sodium hydroxide (NaOH) and 10% v/v cell suspension was added to give cellular collagen at a series of cell densities (0.5–1.5 × 10^6^ cells/ml of gel). These cell seeding densities were based upon the range used within NRCs (Coy et al., 2020; Georgiou et al., 2015; O'Rourke et al., 2018) (Table 1).

**Table 1:** Main parameters for in vitro experiments

	Experimental values
Cell seeding density	$0.5\times 10^{6},0.78\times 10^{6},1.5\times 10^{6}\;\text{cells}/\text{ml}$
Equivalent cell seeding density after stabilization	$20\times 10^{6},31\times 10^{6},60\times 10^{6}\;\text{cells}/\mathrm{ml}$
Oxygen concentration	1%, 3%, 7%, or 21%
Glucose concentration	25 mM
Duration	24 h

Next, 240 μl of the cellular collagen mixture was added to individual wells of a 96 well plate and the gels were allowed to set at 37°C for 15 min. Using RAFT absorbers (Lonza Bioscience) the gels were stabilized using plastic compression for 15 min, a process whereby a biocompatible absorbent material is placed upon the gel and absorbs interstitial fluid to generate a dense, robust hydrogel (Brown et al., 2005). The resulting compressed gels were then immersed in culture medium and incubated at 37°C in a humidified incubator for 24 h under different oxygen concentrations, chosen to reflect the range of oxygen concentrations in which cells would reside in vivo.

### 2.3 Low oxygen 3D cell culture and oxygen monitoring

A hypoxia workstation and incubator (HypoxyLab, Oxford Optronix) was used for experiments requiring low oxygen conditions. Cell culture medium for hypoxic experiments was conditioned to the target oxygen concentration (1%, 3%, or 7%), which encompasses the rage of endoneurium in vivo measurements, for 2 h before use. Cellular collagen constructs were cultured in the HypoxyLab at the desired oxygen concentration for 24 h. In situ dissolved oxygen within the constructs was measured using the integrated OxyLite™ (Oxford Optronix) monitoring system. Fiber‐optic oxygen probes (Oxford Optronix) were inserted into the middle of the constructs. The sensor probes were set to continuously measure oxygen partial pressure (5 samplings/min). The results were recorded using Labview software (National Instruments). Results are presented as partial pressure values in mmHg (e.g. 7.6 mmHg corresponds to 1%).

### 2.4 CellTiter‐Glo assay

Metabolic activity was examined by measuring adenosine triphosphate (ATP) as an indicator and generating a luminescent readout, using the CellTiter‐Glo® 3D Assay (Promega). Based on the manufacturers protocol, a volume of reagent equal to that of the culture medium was added to each experimental well and following a 30‐min incubation at room temperature, 200 μl from the assay solution were transferred to a microplate and luminescence was quantified on a plate reader (Flx800, BioTek). The metabolic activity of cells was determined in culture by measuring the intensity of luminescence signals after 24 h.

### 2.5 Live/Dead assay

To assess cell viability, cultures were stained using Syto 21/Propidium Iodide (PI) (Sigma Aldrich). Syto 21 is a green, fluorescent nucleic acid stain that exhibits bright, green fluorescence upon binding to nucleic acids in both live and dead cells. In comparison, PI, which exhibits red fluorescence, cannot permeate viable cells as it reaches the nucleus by passing through disordered areas of dead cell membrane. Thus, using both dyes allows for the simultaneous staining of viable and dead cells.

For the Syto 21/PI staining, the medium was removed from the gels, which were then washed three times with 200 μl of medium (37°C). Subsequently, 200 μl of Syto 21/PI solution (1:1000 dilution) was added and the plates were incubated for 15 min at 37°C before removing the Syto‐21/PI solution. The gels were then washed briefly with 200 μl of culture medium. Finally, an additional 200 μl of culture medium was added to each gel before image acquisition. Images were visualized using a confocal microscope (Zeiss‐LSM710, Carl Zeiss) with ×20 phase‐contrast water immersion objective. High‐throughput quantification of cell viability from 3D image stacks by adapting a readily available ImageJ protocol was performed.

The spatial distribution of viable cells within the constructs was also estimated. From the 3D image stacks three different zones that correspond to the top, middle, and bottom of the gels were identified and the mean value of live and dead cells per zone was calculated by analyzing seven stacks per zone.

### 2.6 Glucose detection assay

Glucose consumption was quantified by an enzymatic assay (Glucose [HK] Assay Kit, GAHK20, Sigma Aldrich) according to the manufacturer's guidelines. Briefly, after 24 h incubation of the cellular gels, the supernatants were collected for further analysis. The reconstituted reagent was added to each sample and the resulting solution was incubated for minutes at room temperature. During that time, glucose was phosphorylated by ATP, a reaction which was catalyzed by hexokinase. Glucose‐6‐phosphate (G6P) was then catalytically oxidized to 6‐phospho‐gluconate in the presence of oxidized nicotinamide adenine dinucleotide (NAD). Due to this oxidation, an equimolar amount of NAD was reduced to NADH, thereby changing the optical absorbance of the sample. The consequent increase in absorbance was measured at 340 nm and was directly proportional to glucose concentration.

### 2.7 VEGF release

The concentration of secreted vascular endothelial growth factor‐A (VEGF‐A) post 24 h incubation was determined by an enzyme‐linked immunosorbent assay (ELISA). The cell medium supernatant from the gels was collected, stored at −20°C and later analyzed with a VEGF‐A sandwich ELISA kit (human and rat VEGF‐A kits, RayBiotech) according to the manufacturer's protocols.

### 2.8 Experimental data analysis

Normality was determined using a Shapiro‐Wilk test. Two‐way statistical analysis of variance (ANOVA) was conducted, followed by Bonferroni's multiple comparison test.

### 2.9 Cell‐solute mathematical model

**Figure 1:**
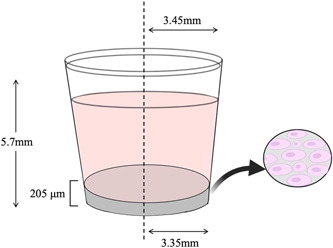
Cell culture well schematic.

The cell‐solute model is comprised of a set of continuous diffusion‐reaction equations that describe the interactions between oxygen (c), glucose (s) and VEGF (v) concentrations and the cell population (n), within the in vitro well setup. These variables were selected as they reflect the potential effect of the local microenvironment on the viability of seeded cells and the expression of VEGF, both of which are fundamental for nerve regeneration and vascular regeneration. We consider the gel and the medium above within the well geometry (Figure 1) as continuous materials with effective uniform properties. In the following sections, we start by describing the model equations in the gel and culture medium, followed by initial and boundary conditions.

We consider the transport of oxygen in the gel to be driven by molecular diffusion, modeled using Fick's first law, and assume that cells metabolize oxygen following Michaelis–Menten kinetics, as is commonly used in the literature for conditions where oxygen is the limiting factor (Haselgrove et al., 1993; Huang et al., 2011; Magliaro et al., 2019; Zhong et al., 2018).

(1) $$\partial_{t}c_{g}=D_{c,g}\nabla^{2}c_{g}-M_{c}n\frac{c_{g}}{c_{g}+\bar{c}},$$

where $c_{g}$ represents the oxygen concentration in the gel, n the local cell density in the gel, $D_{c,g}$ the diffusion coefficient of oxygen in the gel, $M_{c}$ the maximum oxygen consumption rate by the cells, and $\overset{®}{c}$ the oxygen concentration for which oxygen consumption by the cell is half its maximal value. Oxygen is considered to diffuse freely in the medium so that

(2) $$\partial_{t}c_{m}=D_{c,m}\nabla^{2}c_{m},$$

where $c_{m}$ represents the oxygen concentration in the medium and $D_{c,m}$ is the diffusion coefficient of oxygen in the medium.

Next, the equation that governs glucose concentration within the gel can be written as

(3) $$\partial_{t}s_{g}=D_{s,g}\nabla^{2}s_{g}-M_{s}n\frac{s_{g}}{s_{g}+\bar{s}}\left(1+A\frac{\bar{c}}{c_{g}+\bar{c}}\right),$$

where $s_{g}$ represents the glucose concentration in the gel. Here, $D_{s,g}$ is the diffusion coefficient of glucose in the gel, $M_{s}$ the maximum glucose consumption rate by the cells, and $\overset{®}{s}$ the glucose concentration for which glucose consumption by the cell is half its maximal value. Glucose consumption by cells is assumed to follow Michaelis–Menten kinetics (Aubert & Costalat, 2005; Dienel et al., 2017) modified with an additional term to capture anaerobic metabolism given the anticipated local oxygen conditions, $\frac{\bar{c}}{c_{g}+\bar{c}}$. The functional form of this new term is based on supplemental consumption of glucose in anaerobic conditions happening around the same oxygen level as the weakening of the oxygen metabolic reaction ($\overset{®}{c}$) but being negligible for high oxygen concentration conditions. This extra term enables the impact of anaerobic metabolism to be captured while only introducing one new parameter into the model.

Similar to oxygen, we assume that glucose diffuses freely in the medium so that:

(4) $$\partial_{t}s_{m}=D_{s,m}\nabla^{2}s_{m},$$

where $s_{m}$ represents the glucose concentration in the medium and $D_{s,m}$ is the diffusion coefficient of oxygen in the medium.

Next, we consider the VEGF as an unstable molecule secreted by cells. Although vascular endothelial growth factors are a family of polypeptides, in this study we focus on modeling VEGF‐A, which is considered the key mediator of angiogenesis (commonly referred to as VEGF). We describe the VEGF concentration in the gel by:

(5) $$\partial_{t}v_{g}=D_{v,g}\nabla^{2}v_{g}-Kv_{g}+G(c_{g},n),$$

where $v_{g}$ represents the VEGF concentration in the gel, $D_{v,g}$ is the diffusion coefficient of VEGF in the gel, K the VEGF degradation rate and G is the production rate of VEGF. Given that the production rate of VEGF by dCTX0E03 cells is not defined in the literature, we used the experiments presented in Figure 4 to define a production rate of VEGF that considers upregulation under low oxygen conditions. The final relationship describing the dependence of VEGF production on the underlying local environment is given by

(6) $$G=n\left(\alpha c_{g}+\beta \left(e^{-\left(\frac{c_{g}}{c_{\tau }}+\frac{n}{n_{\tau }}\right)}\right)\right),$$

where α represents the baseline VEGF production rate and β represents the VEGF production rate depending on oxygen. Further $c_{\tau }$ is the hypoxic threshold for VEGF production and $n_{\tau }$ represents a crowding factor for the cells. VEGF is also assumed to diffuse freely in the medium:

(7) $$\partial_{t}v_{m}=D_{v,m}\nabla^{2}v_{m}-Kv_{m},$$

where $v_{m}$ represents the VEGF concentration in the medium and $D_{v,m}$ the diffusion coefficient of VEGF in the medium.

Finally, the viable cell density is determined by the balance of cell proliferation and death, along with cell migration. In collagen gels, however, cell migration is negligible on the short timescales considered here (Ardakani et al., 2014) and thus neglected. In addition, CTX0E03 cells are conditionally immortalized and thus, do not proliferate in the absence of 4‐OHT. We describe cell death as an increasing function of cell density, to represent competition for space, and a decreasing function of oxygen and glucose concentration, to represent competition for nutrients so that

(8) $$\partial_{t}n=-n\left(\delta_{0}+\delta_{c}\left(\frac{\bar{c}}{c_{g}+\bar{c}}\right)+\delta_{s}\left(\frac{\bar{s}}{s_{g}+\bar{s}}\right)\right),$$

where $\delta_{c}$ controls the oxygen‐related death, $\delta_{s}$ controls the glucose‐related death $\delta_{0}$ encompasses all other interactions. The rationale behind the choice of oxygen and glucose‐related deaths terms is exactly the same as that for anaerobic consumption (Equation 3).

### 2.10 Well geometry and boundary conditions

The model consists of an axisymmetric 2D geometry (rotational symmetry along the vertical axis of the well), that represents the well of a 96‐well plate and is composed of two domains: (i) the cell‐seeded collagen gel at the base of the well, and (ii) the volume of culture medium above it (Figure 1). Conditions are imposed at the boundaries to capture the geometrical constraints and relevant transport characteristics of the setup. At the boundary between the cellular gel and the medium, we assume continuity of concentration and flux for oxygen, glucose, and VEGF. Zero flux boundary conditions are imposed for oxygen at the bottom and the sides of the well, whereas the concentration of oxygen on the medium‐air interface was set as constant and equal to the ambient oxygen as prescribed during the experiments. For VEGF and glucose, zero flux conditions were set at the bottom, sides and air interface. The initial oxygen concentration in the gel was set at $c_{g}\left(r,z,t=0\right)=12\%,$ due to preconditioning of the cell culture medium. Based on the experimental data, the initial glucose concentration at the medium was set as $s_{g}\left(r,z,t=0\right)=25$ and 1.25 mM in the gel, the initial VEGF concentration was set at v(r,z,t=0)=0, and the initial cell density was varied to match the experimentally‐imposed seeding conditions.

### 2.11 Parameter values

Initially, the bounds of the parameters included in the model equations were informed based on literature values (see Table 2). For some parameters, literature was either scarce or conflicting and often from a range of cell types and culture conditions that do not represent the specific setup here. Thus, bounds for some parameters were chosen based on our own experimental observations and conditions.

**Table 2:** Initial parameter range

	**Nominal value and approximated bounds**
**Cell density parameters**	
Maximal cell density ($n_{\max})$	$n_{\max}=77\times 10^{6}$ cell/ml
Proliferation rate constant	0
Baseline cell death rate constant ${(\delta }_{0g})$	[3.3 × 10^−7^, 1.1334 × 10^−5^ 1/s] (Chung et al., 2006; Coy et al., 2020)
Hypoxic cell death rate constant ${(\delta }_{c})$	^a^[1.7 × 10^−8^, 4 × 10^−6^ ]1/s
Glucose deprivation‐induced death rate constant ($\delta_{g})$	^a^[1.7 × 10^−8^, 4 × 10^−6^ ]1/s
**Oxygen concentration parameters**	
Diffusion coefficient for oxygen in medium ${(D}_{c_{m}})$	$D_{c_{m}}=$ 2.62 × 10^−5^ [1 × 10^−5^, 4 × 10^−5^] cm^2^/s (Han & Bartels, 1996)
Diffusion coefficient for oxygen in gel ${(D}_{c_{g}})$	[1 × 10^−6^, 4 × 10^−6^] cm^2^/s (Cheema et al., 2012)
Concentration at which oxygen consumption is ½ maximal ($\overset{®}{c})$	[$6.66\times 10^{-9},4\times 10^{-8}$] mol/ml (Coy et al., 2020) (0.5% O_2_)
Maximal rate of oxygen consumption $\left(M_{c}\right)$	^b^[$1\times 10^{-18},7.7\times 10^{-16}$] mol/cell/s (Herculano‐Houzel, 2011; McMurtrey, 2015; Streeter & Cheema, 2011; Wagner et al., 2011)
**Glucose concentration parameters**	
Diffusion coefficient for glucose in medium ($D_{s_{m}})$	[5.65 $\times 10^{-6},$ 1.09 $\times 10^{-5}$] cm^2^/s (Shipley et al., 2009; Suhaimi et al., 2015)
Diffusion coefficient for glucose in gel ($D_{s_{g}})$	[0.23 $\times 10^{-6},$ 1.51 $\times 10^{-6}$] cm^2^/s (Cochran et al., 2006; Wu et al., 2005)
Concentration at which glucose consumption is 1/2 maximal ($\overset{®}{s})$	[2,10] mM (Barros et al., 2007; Duarte et al., 2009; Van Zijl et al., 1997)
Maximal rate of Glucose consumption ${(M}_{s})$	[5 × 10^−17^, 2.2 × 10^−16^] mol/cell/s (Gu et al., 2016; McMurtrey, 2015)
Anaerobic threshold for glucose consumption (A)	N/A
**VEGF concentration parameters**	
Diffusion coefficient for VEGF in medium ($D_{v_{m}})$	[$1.3\times 10^{-6},2\times 10^{-6}$] cm^2^/s (Mac Gabhann et al., 2005; Mac Gabhann et al., 2007)
Diffusion coefficient for VEGF in gel ($D_{\mathrm{vg}})$	[2.9 × $10^{-7},$ 1.13 × $10^{-6}$] cm^2^/s (Chen et al., 2007; Köhn‐Luque et al., 2013; Wang et al., 2020)
VEGF degradation rate (K)	[2.67 × 10^−6^, 1.28 × 10^−4^] 1/s
Hypoxia threshold for VEGF secretion ($c_{\tau }$)	a[0.1, 2] %O_2_
VEGF crowding factor ($n_{\tau })$	a[1, 60] $\times 10^{6}$ cell/ml
Baseline VEGF secretion rate at low oxygen (a)	N/A
Baseline VEGF secretion rate based on oxygen (β)	N/A

^a^ Based on experimental observations.

^b^ Bounds of parameter were adjusted to account for decreased oxygen consumption rate in 3D culture systems (Magliaro et al., 2019).

### 2.12 Sensitivity analysis

The model defined by Equations (1)–(9), while built with minimal components, still includes 19 parameters, excluding initial and boundary conditions. The large number of model parameters compared to the relatively small data set, due to the limited spatial and temporal resolution of the in vitro model, leads to an underdetermined system. To help regularize the problem, we performed a sensitivity analysis to prioritize the importance of parameters in predicting a relevant quantity of interest, chosen here as the average concentrations and cell density in the gel,

(9) $$\langle x_{g}\rangle (t=24h)=\frac{1}{V_{g}}{∭}_{V_{g}}x_{g}dV,$$

where $x_{g}$ represents the local concentration of the different species in the gel and $V_{g}$ the volume of the gel. By combining this prioritization exercise with existing knowledge of the different parameters, we can define realistic intervals for each parameter value. Such intervals are then explored during the optimization procedure (small intervals for impactful parameters, large intervals for minor parameters) by comparing model predictions against experimental measurements.

Multiple approaches exist to perform sensitivity analysis (Saltelli et al., 2004). Given the size of the parameter set we selected the Morris Screening Method (Morris, 1991) which qualitatively evaluates the global sensitivity of each parameter, including coupling and nonlinearity, using a set of elementary effects. More specifically, we use the implementation proposed by Campolongo et al., who developed an optimized parameter sampling algorithm for sensitivity analysis with decreased computational cost (Campolongo et al., 2007).

Input values of the variables of interest are determined based on a sampling algorithm which starts at randomly selected points in the *k*‐dimensional space and creates a trajectory through all the *k*‐dimensions. First, the elementary effects (*EE*_*i,j*,_ *i* = 1, …, *r, j* = 1, …, *k*) used for the Morris screening test are individually computed for each trajectory and each variable of interest.

$$EE_{i,j}=\frac{Y\left(X_{1},X_{2},\text{…},X_{i}+\Delta_{i},\text{…},X_{k}\right)-Y\left(X_{1},X_{2},\text{…},X_{i},\text{…},X_{k}\right)}{\Delta_{i}}=\frac{Y\left(X+\Delta e_{i}\;\right)-Y(X)}{\Delta }.$$

An elementary effect can be computed if and only if $X+\Delta e_{i}$ is still in the parameter range. This means that there are $p^{k-1}(p-\Delta \left(p-1\right))$ elementary contributions. *E* $E_{i,j}$ values are then used to calculate the final sensitivity measures such as the mean absolute value of the elementary effect and the standard. More specifically,

$$\mu_{i}^{*}=\frac{1}{r}\sum \left|EE_{i,j}\right|$$

and

$$\sigma_{i}=\sqrt{\frac{1}{r}\sum {\left(EE_{i,j}-\frac{1}{r}\sum EE_{i,j}\right)}^{2}},$$

with $\mu_{i}^{*}$ being used to detect input parameters that have an overall influence on the output, and σ being used to detect input parameter involved in interactions or nonlinearities.

### 2.13 Optimization

We seek to minimize the difference between the model predictions and experimental measurements by defining the underlying parameters. Given the size of the parameter set, we choose to perform a global, heuristic optimization using a particle swarm method (Kennedy & Eberhart, 1995). The particle swarm algorithm seeks to find an approximate solution to the equation:

(10) $$\underset{\theta }{\mathrm{argmin}}J\left(\theta ,\left\{X\right\}\right),$$

where θ is the vector of parameters, {X} the set or subset of species of interest (i.e., oxygen, glucose, VEGF, and cell density) and where J describes the cost function

(11) $$J\left(\theta ,\{X\}\right)=\sum_{\left\{X\right\}}\frac{1}{\Omega_{X}}{||F_{X}\left(\theta ,\sigma_{IB}\right)-Y_{X}\left(\sigma_{IB}\right)||}_{L_{1}},$$

which when minimized corresponds to minimizing the average difference between experimental measurements and simulations for a given set of species. Thus $\Omega_{X}$ is the size of the experimental set for species X, $F_{X}\left(\theta ,\sigma_{IB}\right)$ the vector containing the corresponding predicted values for a given vector of parameter value θ and vector of initial and boundary values $\sigma_{IB}$, and $Y_{X}\left(\sigma_{IB}\right)$ the vector corresponding to the corresponding experimental measurements. This approach has the advantages of avoiding possible local minima, considering the hierarchy between parameters, and imposing very few constraints on the regularity of the cost function itself.

### 2.14 Numerical simulations

The model (Equations 1–9) was solved numerically using finite volume methods in Python 3.7. Given the rotational symmetry of a culture well, the model is solved in two‐dimensional (radial and axial). However, since the slope of the well geometry is small (<0.2%), variations in the radial direction can also be considered negligible compared to the axial ones, effectively rendering the model one‐dimensional. As for the axial direction, we devised a nonuniform two‐part mesh corresponding to gel and medium with a change in mesh cell density at the interface between the two domains. This is done to allow a finer resolution in the gel where gradients are steeper, while still capturing the interface between gel and medium exactly.

Based on a mesh convergence analysis, N=100 mesh cells (80% in the medium and 20% in the gel) with a timestep Δt=100 s enabled gradient fields to be sufficiently resolved in both domains. Further increasing the spatial or temporal resolution resulted in at most in ~1% change for the average concentration in the gel after 24 h. Next, the Morris sensitivity analysis was run using the open source library SALib (Herman & Usher, 2017) using the intervals presented in Table 2 with 40 trajectories on a four‐level grid.

Finally, the particle swarm optimization was performed using another open source library, PySwarm (Miranda, 2018), with three meta‐parameters (two acceleration coefficients c1, c2 to control the individual and collective behavior, and one inertia coefficient *w* to control a history effect). The implementation was split in two separate steps (1) optimization of the oxygen, glucose and cell density related parameters (which are mutually coupled) (2) optimization of the VEGF related parameters (independent of the other species). For each step, we use 20 particles and 1250 samples leading to 25,000 simulations. For step 1, c1 = 2, c2 = 0.2, *w* = 0.6 whereas for step 2, c1 = 2, c2 = 0.2, *w* = 0.7 yielded the optimum results and met the appropriate convergence criteria (as defined in (Jiang et al., 2007)). During step (1) we compute the average oxygen in the gel every 0.5 h for 24 h for: (a) ambient oxygen concentrations $c_{m}(z=0)=$ 1%, 3%, and 7% and for an initial cell density n(t=0)=  60 ($\Omega_{c}=144)$, (b) average glucose concentration in the culture medium after 24 h for $c_{m}(z=0)=$ 1%, 3%, 7%, and 19% and for initial cell densities n(t=0)= 20, 31, 60 ($\Omega_{s}=12)$, (c) average cell density in the gel after 24 h for 1%, 3%, 7%, and 19% ambient oxygen concentrations and for initial cell densities n(t=0)= 20, 31, 60 ($\Omega_{n}=12)$. For consistency in calculating the cost function (Equation 11), all results were nondimensionalised using the initial concentration for each species. Similarly, for step (2), we compute the average VEGF concentration in the culture medium for ambient oxygen concentrations $c_{m}(z=0)=$ 1%, 3%, 7%, and 19% and for initial cell densities n(t=0)= 20, 31, 60 ($\Omega_{v}=12)$.

## 3 RESULTS

### 3.1 Viability and metabolic activity of dCTX0E03 cells under different oxygen conditions

**Figure 2:**
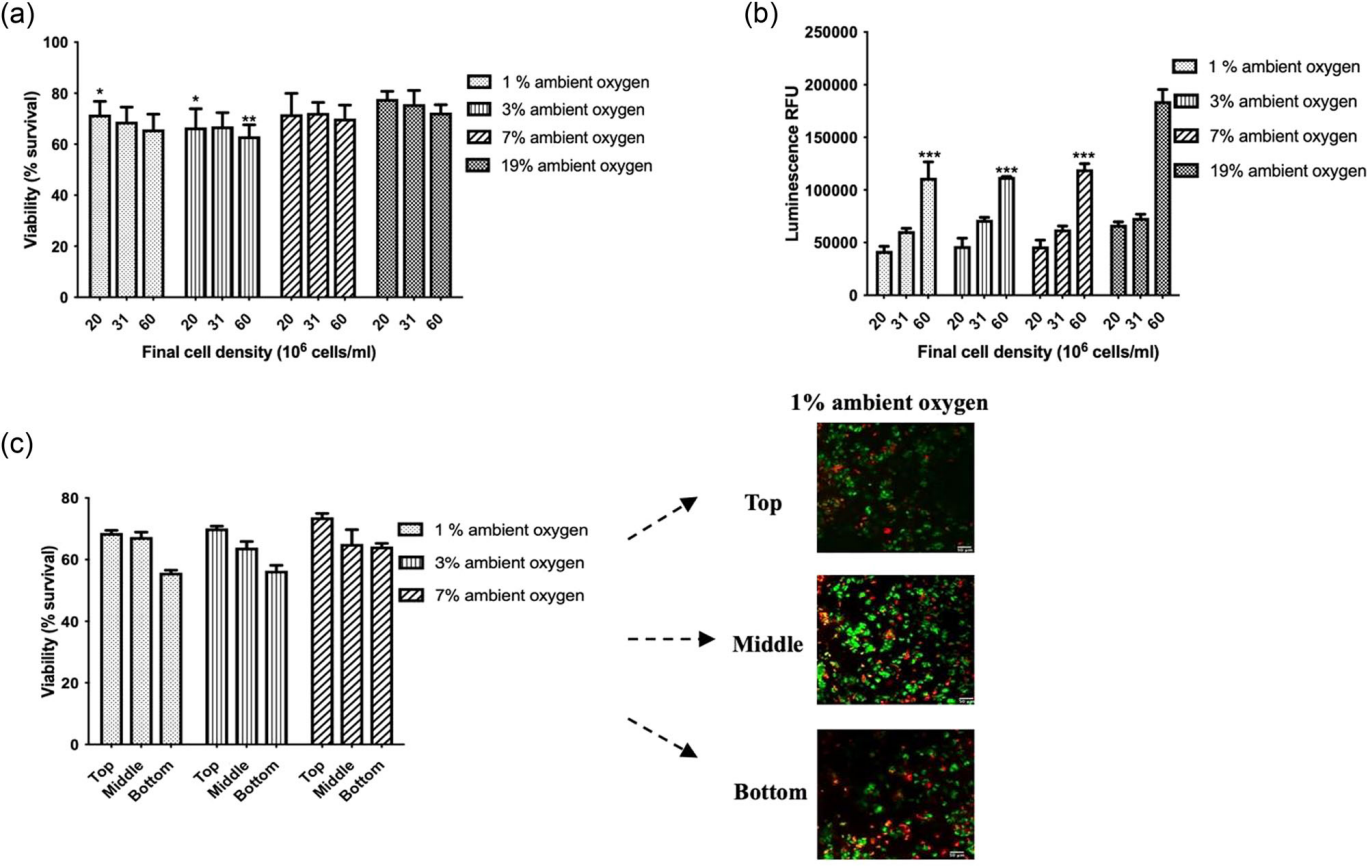
dCTX0E03 cell survival and metabolic activity in stabilized collagen gels exposed to different oxygen conditions for 24 h. (a) Cell viability was calculated using live/dead staining and analysis of obtained optical sections. Syto21 was used to label all cells and propidium iodide to label dead cells. (b) Metabolic activity was assessed using the 3D CellTiter‐Glo assay. Data expressed as means ± SEM. Significance levels were *p < 0.033; **p < 0.002; and ***p < 0.001 compared with normal culture conditions (19%). (c) Spatial variability in the viability of dCTX0E03 cells in stabilized collagen gels exposed to different oxygen conditions for 24 h (60 × 10^6^ cells/ml density after stabilization). Data expressed as means ± SEM (*n* = 4 independent repeats, three samples per condition).

The potential use of cellular NRCs for the treatment of PNI is dependent on the ability of encapsulated cells to remain viable and maintain their therapeutic effects. Therefore, the proportion of viable cells and their metabolic activity were evaluated. Figure 2a,b illustrate the survival of target cells under low oxygen conditions. Reduced oxygen availability seemed to cause impairment in dCTX0E03 survival and metabolic activity, with the effect being more pronounced at the highest seeding densities. There was up to 10% reduction in viability and up to 26% reduction in metabolic activity after incubation at 1% O_2_ for 24 h, when compared with normoxic conditions. Differences in cellular responses can also be observed between the higher oxygen tensions, although they are not as pronounced. Finally, cell death appears to be higher at high cell seeding density conditions, possibly due to competition for available nutrients.

We also explored the spatial distribution of viable cells within the constructs (Figure 2c). Results indicate that areas of greater viable cell density occur at the top of the gels, correlating with highest oxygen concentrations at the air interface (and lowest at the well base which is furthest from the oxygen source) (Cheema et al., 2007).

### 3.2 Oxygen consumption characteristics in 3D constructs

**Figure 3:**
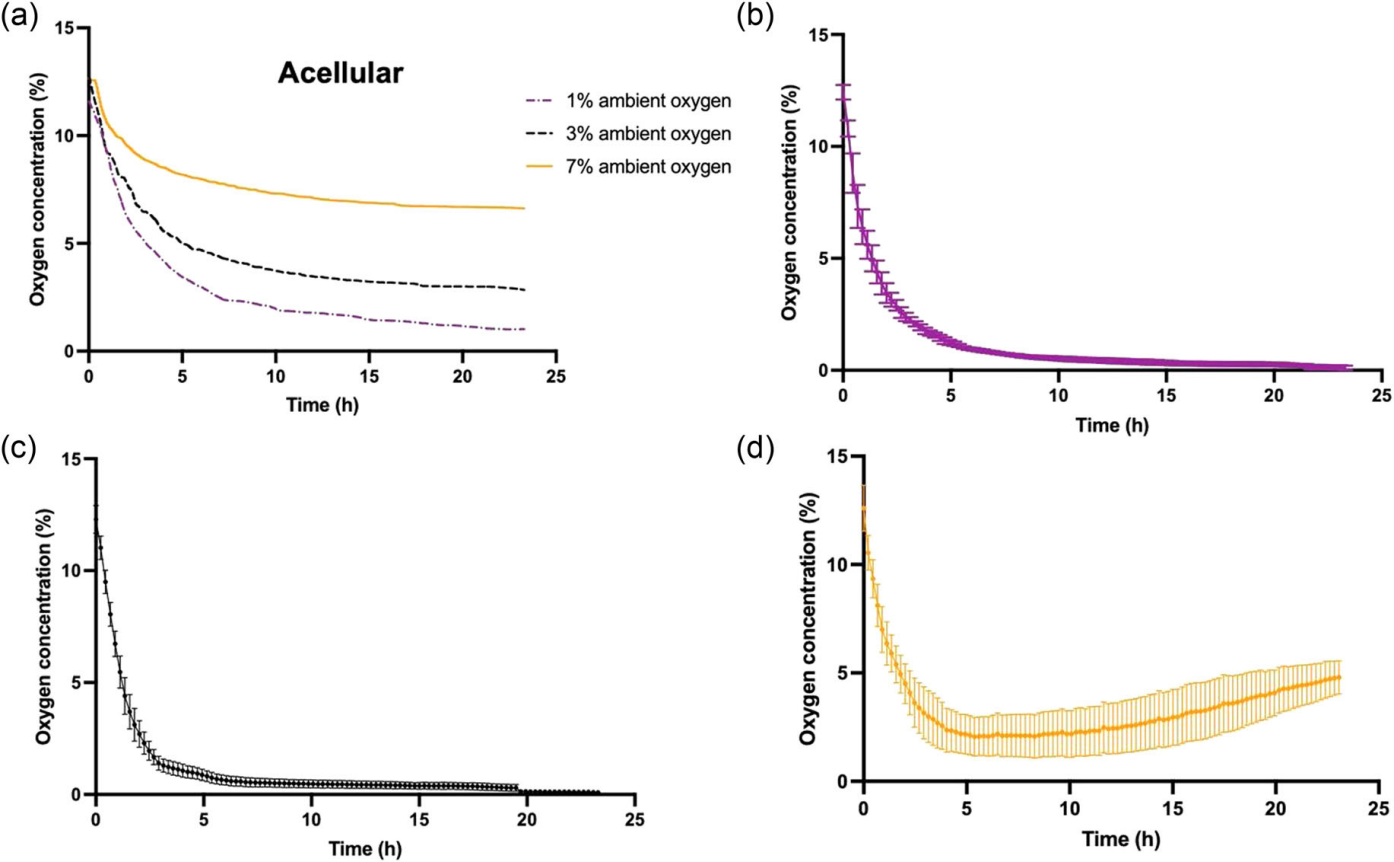
Oxygen levels in the center of (a) acellular (*n* = 1) or dCTX0E03‐seeded constructs (60 × 10^6^ cells/ml density after stabilization) at (b) 1% oxygen, (c) 3% oxygen, (d) 7% oxygen. Time zero refers to the time point when the probe was positioned in the gel. Data expressed as means ± SEM (*n* = 3).

Figure 3 displays the temporal changes in the oxygen concentrations measured at the center of cellular stabilized collagen constructs cultured under different ambient oxygen levels. Studies on acellular constructs (Figure 3a) show that the oxygen concentration in the gel equilibrated to ambient levels within 5 h. Any differences in these profiles for cellular gels must be due to cellular metabolism.

Cellular constructs exhibited time‐dependent oxygen depletion in their core (Figure 3b–d). There was a rapid fall of oxygen toward approximately steady‐state values, with the rates being affected by the ambient oxygen level. The lowest ambient oxygen concentration of 1% caused the steeper gradients for collagen gels with dCTX0E03 cells. Interestingly, the oxygen concentration appears to reincrease after 12 h for cellular constructs cultured at 7%. Between 0 and 12 h, dCTX0E03 cells cultured at 7% oxygen exhibit similar consumption characteristics as at other oxygen levels, namely a fast, initial decrease of the oxygen concentration, followed by much lower decrease rates. After 12 h a recovery is observed which could be attributed to a shift in the equilibrium between oxygen metabolism and supply. As a proportion of the embedded cells die, total oxygen consumption decreases which in turns leads to a reincrease in local oxygen levels.

### 3.3 Functional analysis of dCTX0E03 cells under different oxygen conditions

**Figure 4:**
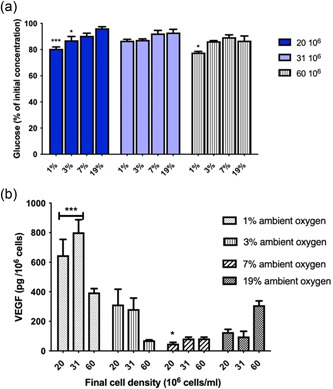
Functional analysis of dCTX0E03 cells under different oxygen conditions. (a) Glucose consumption by dCTX0E03 cells seeded in collagen and cultured under a range of ambient oxygen concentrations for 24 h. Glucose concentration was quantified using a biochemical assay. Data expressed as means ± SEM (*n* = 4 independent repeats, three samples per condition). (b) VEGF release from dCTX0E03 cells seeded in collagen and cultured under a range of ambient oxygen concentrations for 24 h. VEGF concentration was measured via ELISA. Original values are divided by the initial cell seeding density. Data expressed as means ± SEM (*n* = 4 independent repeats, three samples per condition). Significance levels were *p < 0.033; **p < 0.002; and ***p < 0.001 compared to normal culture conditions (19%).

Oxygen bioavailability is also directly linked to energy homeostasis. Lower oxygen levels compromise the function of mitochondria in generating cellular energy currency, ATP, through oxidative phosphorylation, which is the most efficient way of producing ATP from glucose. This causes cells to rely on glycolytic ATP generation. Figure 4a demonstrates glucose consumption during the experiments. The glucose utilization rate was higher at low oxygen conditions. The decrease was also more pronounced at higher cell seeding densities.

Finally, as illustrated in Figure 4b, subjecting cells to physiological stress through oxygen deprivation stimulates and subsequently increases the expression of VEGF. VEGF release was affected by the local oxygen levels and cell seeding density, however, the relationship between them was not straightforward. Activation of VEGF expression by hypoxia‐induced stress was more prominent at mild to severe hypoxia (1%–3%). For 1%–3% ambient oxygen concentrations, upregulation of VEGF release appears to reach maximum levels at $n_{0}=20\times 10^{6}$ and $n_{0}=31\times 10^{6}$ cells/ml, where cells were found to be more active. This trend was reversed for mildly hypoxic and normoxic conditions.

### 3.4 Mathematical model

**Figure 5:**
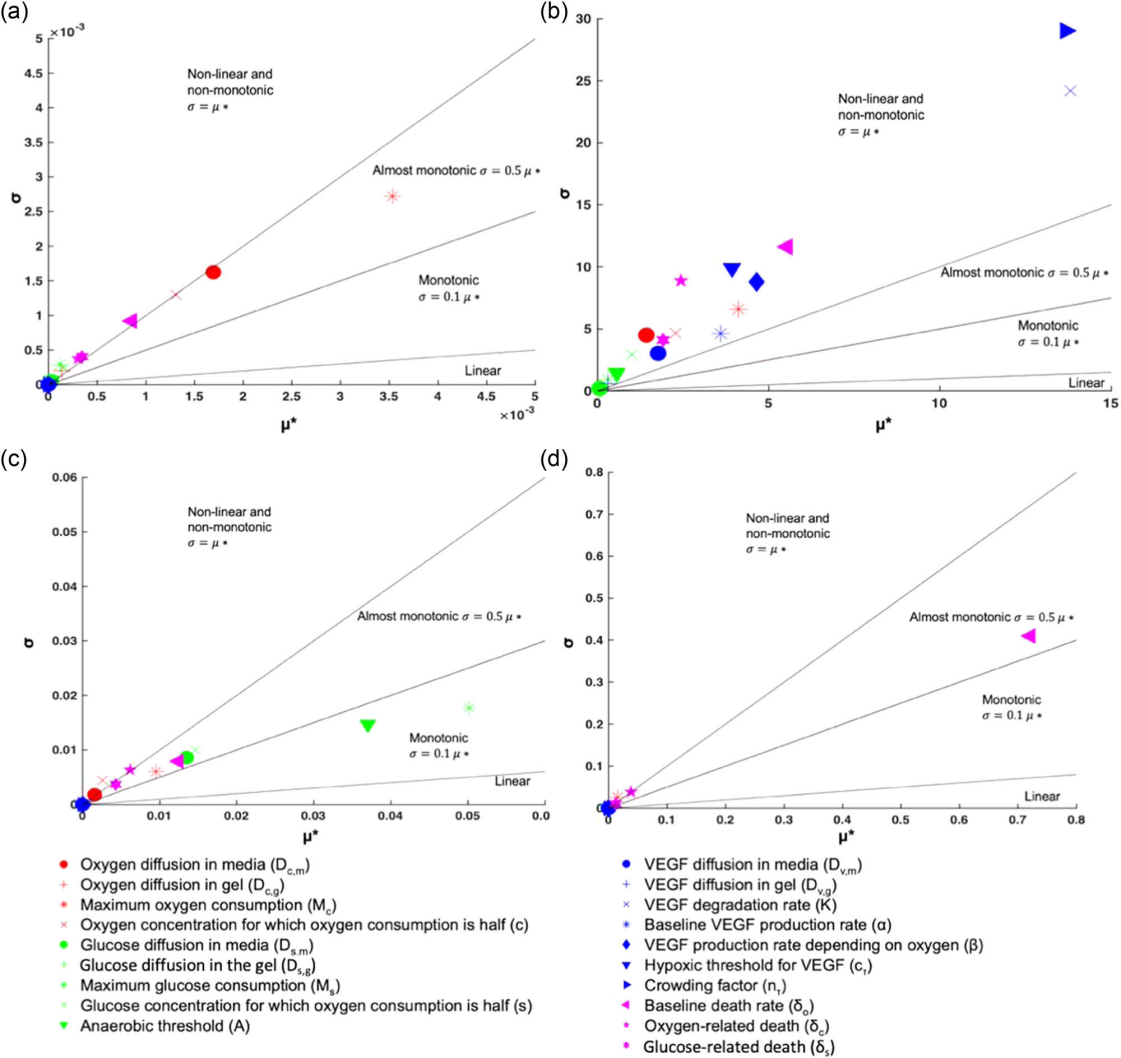
Morris sensitivity analysis results based on final (a) oxygen, (b) VEGF, (c) glucose, (d) cell density values in the center of gel after 24 h. Each point represents the mean absolute value μ* (*x*‐axis) and standard deviation *σ* (*y*‐axis) of the elementary effect of each parameter; the first is used to identify which input parameters have an overall influence on the output (i.e., oxygen, VEGF, glucose, cell density) and the latter can help identify which input parameters are involved in interactions or nonlinearities.

We derived a cell‐solute model for a well geometry, which needed to be further parametrized for the specific cell type used. The sensitivity analysis enabled the prioritization of parameters that contribute most to variation in model predictions as summarized in Figure 5, where the values μ* (*x*‐axis) and *σ* (*y*‐axis) capture the impact of each model parameter on output predictions, and identify which parameters contribute to couplings or nonlinear effects, respectively. Based on previous literature the individual parameters can also be classified in terms of (non‐) linearity, (non‐) monotony based on their individual *σ*/μ* ratio (Garcia Sanchez et al., 2014).

The oxygen concentration is mainly affected by the diffusion coefficient in the media, the oxygen concentration for which consumption is half‐maximal, the maximum rate of oxygen consumption, the oxygen‐related death rate, and the baseline death rate (Figure 5a). The maximal rate of oxygen consumption has the strongest influence on model predictions and interacts most with other parameters. For the VEGF concentration, most parameters exhibit a nonlinear influence on and/or interactions with other parameters (*σ*/μ* > 0.5). The final concentration is mostly affected by the crowding factor and VEGF degradation (Figure 5b). The effect of a large group of parameters including VEGF production rate, maximum oxygen consumption rate, and baseline death rate can be considered secondary but nonnegligible. For glucose (Figure 5c), the model output varies nonlinearly but monotonically or almost monotonically with the maximum rate of glucose consumption, anaerobic threshold, glucose diffusion in the medium, and the glucose concentration for which consumption is half of the maximum. The global sensitivity analysis also indicates that the maximum rate of oxygen consumption is a potentially influential parameter in glucose concentration. Finally, the cell viability after 24 h is mostly affected by the baseline death rate (Figure 5d). Those parameters identified as having minimal influence on model outputs were then fixed at the nominal values provided in Table 2 (${n_{\max},D}_{c_{m}},D_{s_{m}},D_{v_{m}}$).

Next, we used a particle swarm algorithm to minimize the difference between model predictions and experimental data, via the choice of the remaining parameters ${(\delta }_{0g},{\delta_{c},\delta }_{g},D_{c_{g}}\overset{®}{,c}M_{c}{,D}_{s_{g}},\overset{®}{s},M_{s},A,D_{\mathrm{vg}},K,c_{\tau },n_{\tau },a,\beta )$, as described in the Methods. Table 3 summarizes the final set of parameters found.

**Table 3:** Final parameter values

	Final value (practical units)	Final value (modeling units)
**Cell density parameters**		
Maximal cell density ($n_{\max})$	$n_{\max}=6.0\times 10^{7}$ cell/ml	$n_{\max}=6.0\times 10^{13}\;\mathrm{cell}/m^{3}$
Proliferation rate constant	0	0
Baseline cell death rate constant ${(\delta }_{0g})$	$\delta_{0g}=3.18\times 10^{-6}$ 1/s	$\delta_{0g}=3.18\times 10^{-6}$ 1/s
Hypoxic cell death rate constant ${(\delta }_{c})$	$\delta_{c}=2.53\times 10^{-6}$ 1/s	$\delta_{c}=2.53\times 10^{-6}$ 1/s
Glucose deprivation induced death rate constant ($\delta_{g})$	$\delta_{g}=5.6\times 10^{-7}$ 1/s	$\delta_{g}=5.6\times 10^{-7}$ 1/s
**Oxygen concentration parameters**		
Diffusion coefficient for oxygen in medium ${(D}_{c_{m}})$	$D_{c_{m}}=2\times 10^{-9}$ m^2^/s	$D_{c_{m}}=2\times 10^{-9}$ m^2^/s
Diffusion coefficient for oxygen in gel ${(D}_{c_{g}})$	$D_{c_{g}}=4.98\times 10^{-10}$ m^2^/s	$D_{c_{g}}=4.98\times 10^{-10}$ m^2^/s
Concentration at which oxygen consumption is 1/2 maximal ($\overset{®}{c})$	$\overset{®}{c}=1.65\times 10^{-8}$ mol/ml (1.24% O_2_)	$\bar{c}=5\text{.}13\;\times \;10^{\text{‐}4}\;\text{kg}/m^{3}$
Maximal rate of oxygen consumption ($M_{c})$	$M_{c}=1.84\times 10^{-18}\;\text{mol}/\text{cell}/s$	$M_{c}=5.88\;\times \;10^{\text{‐}20}\;\;\text{kg}/\text{cell}/s$
**Glucose concentration parameters**		
Diffusion coefficient for glucose in medium ($D_{s_{m}})$	$D_{s_{m}}=9\times 10^{-11}\;m^{2}/s$	$D_{s_{m}}=9\times 10^{-11}\;\;m^{2}/s$
Diffusion coefficient for glucose in gel ($D_{s_{g}})$	$D_{s_{g}}=2.67\times 10^{-10}\;m^{2}/s$	$D_{s_{g}}=2.67\times 10^{-10}\;m^{2}/s$
Concentration at which glucose consumption is 1/2 maximal ($\overset{®}{s})$	$\bar{s}=7.8\;\text{mM}$	$\bar{s}=1\text{.}39\;{\text{kg/m}}^{3}$
Maximal rate of glucose consumption ${(M}_{s})$	$M_{s}=9.8\times 10^{-18}\;\text{mol}/\text{cell}/s$	$M_{s}=1.75\times 10^{-18}\;\text{kg}/\text{cell}/s$
Anaerobic threshold for glucose consumption (A)	A=4.6	A=4.6
**VEGF concentration parameters**		
Diffusion coefficient for VEGF in medium ($D_{v_{m}})$	$D_{v_{m}}=1.32\times 10^{-10}$ m^2^/s	$D_{v_{m}}=1\text{.}32\times 10^{‐10}$ m^2^/s
Diffusion coefficient for VEGF in gel ($D_{\mathrm{vg}})$	$D_{\mathrm{vg}}=4.16\times 10^{-11}$ m^2^/s	$D_{\mathrm{vg}}=4.16\times 10^{-11}$ m^2^/s
VEGF degradation rate (K)	K $=8.37\times 10^{-5}$ 1/s	K $=8.37\times 10^{-5}$ 1/s
Hypoxia threshold for VEGF secretion ($c_{\tau }$)	$c_{\tau }=1.40\times 10^{-8}$ mol/ml (1.08% O_2_)	$c_{\tau }=4\text{.}49\times 10^{‐4}\;\;\;\text{kg}/m^{3}$
VEGF crowding factor ($n_{\tau })$	$n_{\tau }=2.32\times 10^{7}$ cell/ml	$n_{\tau }=2.32\times 10^{13}\;\;\;{\text{cell/m}}^{‐3}$
Baseline VEGF secretion rate at low oxygen (a)	$\alpha =9.24\times 10^{-2}$ pg/cell/s (molO_2_/ml)^−1^ $(1.21\times 10^{-9}$ pg/cell/s/% O_2_)	$\alpha =2.92\times 10^{-21}\;\;\;\text{kg}/\text{cell}/s$ (kgO_2_/m^3^)^−1^
Baseline VEGF secretion rate based on oxygen (β)	$\beta =2.86\times 10^{-7}$ pg/cell/s	$\beta =2.86\times 10^{-22}\;\;\;\text{kg}/\text{cell}/s$

**Figure 6:**
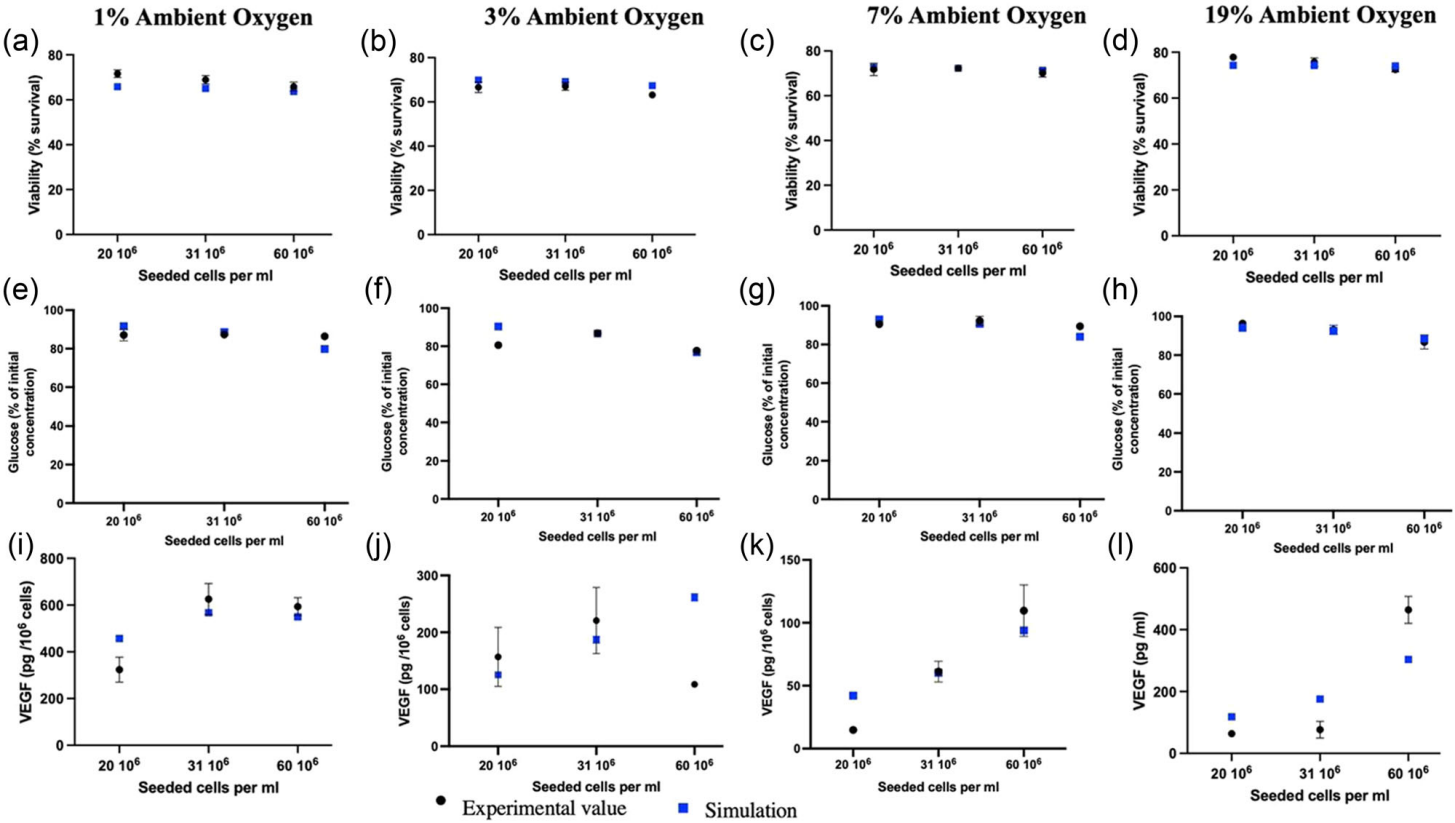
Comparison between experimental and simulation results based on final parameter values of (a–d) cell viability, (e–h) glucose, and (i–l) VEGF in the media after 24 h.

**Figure 7:**
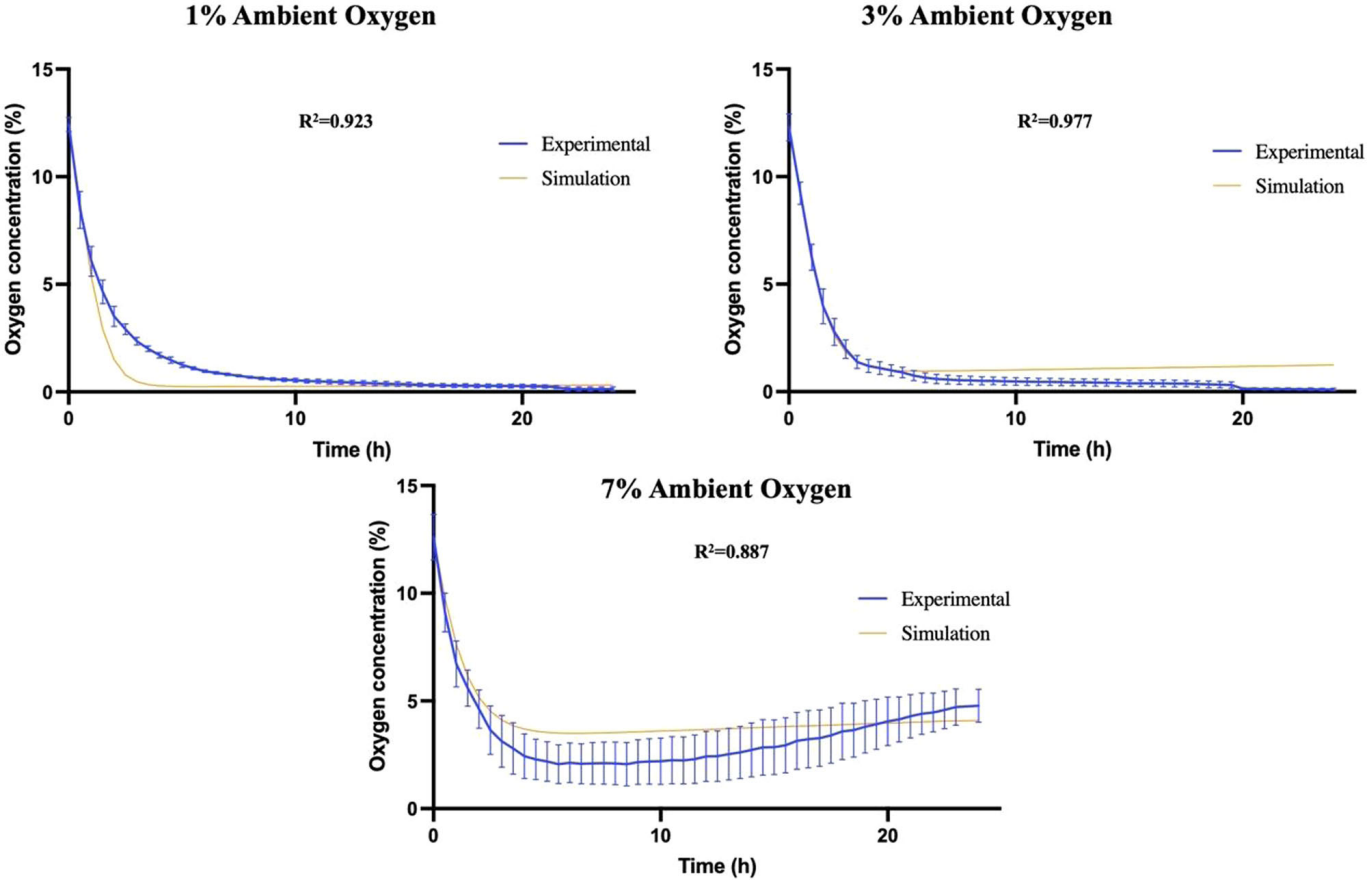
Comparison between experimental and simulation results based on oxygen profiles in the gel after 24 h.

Figures 6 and 7 compare simulation predictions and experimental data for the final optimized parameter value set. Overall, the model replicates the general trends for the viable cell density, nutrient consumption, and VEGF release. The best fit for cell viability is for the 7% O_2_ data set. For the remaining conditions, 1% and 3% O_2,_ the model tends to respectively underestimate and overestimate the mean viable cell density. With regard to glucose consumption, the fit against experimental observations appears to be worse for $n_{0}=60\times 10^{6}$ cells/ml than for the other initial cell densities, but the model predictions closely follow the experimental data points. Regardless of the initial cell seeding density, the simulated concentration of VEGF released into the medium after 24 h is also in good agreement with the corresponding experimental data. The poorest fit was found to be for $n_{0}=60\times 10^{6}$ cells/ml, especially for 3% ambient oxygen concentration. In the case of oxygen consumption, the model qualitatively reproduces the general trend of the experimental data. For instance, in the case of 1% ambient oxygen the broad shape of the oxygen consumption curves matches that of the data, but the rates of decrease appear to be much quicker than the experimental values would suggest is realistic. This could indicate that the oxygen metabolism term requires further refinement in the future.

## 4 DISCUSSION

This study explores the behavior in vitro of therapeutic cells under physiologically relevant oxygen conditions, one of the major determining factors that affect the performance of NRCs in vivo. With regard to oxygen, local supply after implantation is expected to be limited, especially during the first days when neovascularisation has not progressed. dCTX0E03 cells were found to be vulnerable to oxygen conditions they are likely to encounter in situ. However, the reduction of cell viability was not as significant as expected based on previous literature. Extending the duration of the experiments could provide further insights regarding the low long‐term survival upon implantation observed in previous studies (Smith et al., 2012; Stevanato et al., 2009). Moreover, this discrepancy could be associated with the fact that cells adapt by recalibrating their metabolic profile and activating antiapoptotic pathways. Indeed, we observed an increase in the rate of glucose utilization under low O_2_ tension.

Oxygen and glucose deprivation have also been correlated with changes in growth factor release (Mac Gabhann et al., 2007). Our results are consistent with reports that VEGF expression increases under hypoxic conditions; a response that has been linked to neuronal protection and nerve regeneration (Jin et al., 2001; Lee et al., 2016). We also found a correlation between increased glucose consumption and greater VEGF secretion, although this has not been investigated for neural stem cells before.

Next we developed a predictive, cell‐type specific and computationally effective model to describe interactions between dCTX0E03 cells and soluble factors that can be readily used to investigate various nerve repair scenarios. The functional forms of the equations were adapted from previous cell‐solute mathematical models (Chung et al., 2006; Coy et al., 2020; McMurtrey, 2015; Streeter & Cheema, 2011) developed for other cell types. Much of the modeling work done in tissue engineering so far has produced interesting results and generated general hypotheses about the optimization of tissue‐engineered constructs or the tissue culture conditions. However, many of the mathematical models were not benchmarked against a specific or consistent experimental data set or were validated by comparing theoretical simulations with scattered data from multiple sources from the literature, often for different cell types. Here, our focus was to parametrize the model using dedicated in vitro experiments. Aside from the novel set of differential equations that make up the model, we also optimized the parameter values and tailored them to the metabolic and functional characteristics of dCTX0E03s within collagen constructs. The final derived values are mostly within the range reported in the literature (Table 2) for other cell types. One noteworthy exception is the maximal rate of glucose consumption ${(M}_{s})$, which is almost 10 times higher than previously suggested (Gu et al., 2016; McMurtrey, 2015). However, the rate of glucose consumption by differentiated human neural stem cells, in general, is not widely characterized.

Undoubtedly, the model described in this study involves a high degree of simplification of what are in reality complex biological phenomena. Nevertheless, the simulations appear to capture the cellular responses and related trends correctly. Some differences between the model outputs and experimental results were detected, with the largest ones being for $n_{0}=60\times 10^{6}$ cells/ml at 3%, 7% O_2_. To test whether these discrepancies were due to parameter estimation, we ran the PSO algorithm 10 times and confirmed that variability in the predicted parameter values was insufficient to account for the differences between measured and predicted VEGF concentrations (data not shown). This indicates that these differences were due to biological mechanisms that are not captured in the current governing equation set. For instance, the influence of VEGF concentration on the viable cell density was neglected here, even though it has been shown to influence the survival of neural stem cells under hypoxia. Moreover, from the two nutrients examined in this study, only the effect of oxygen was included in the VEGF governing equation.

Finally, another aspect that was ignored when modeling VEGF production and release is the presence of different isoforms. Cells are able to express different VEGF isoforms as part of their physiological processes (Ara et al., 2010; Cain et al., 2014). Still, including multiple species of the same molecule would have drastically increased the complexity of the model and the number of unknown parameters. Each of the isoforms displays unique decay and diffusion characteristics, possibly due to differential collagen binding and proteolytic release (Vempati et al., 2011, 2014). Differential VEGF binding to collagen may be important during the generation of VEGF gradients within the construct and its release in the local microenvironment. Therefore, including this mechanism in the model may improve its ability to predict the temporal and spatial VEGF distributions.

The overall quantitative framework that we developed by combining experimental and theoretical approaches can enable researchers to simulate a wide variety of different engineered tissue configurations and obtain robust predictions about the therapeutic effect of CTX0E03 cells embedded in NRCs. For instance, during the first critical hours upon implantation, therapeutic cells adapt to their environment by rapidly consuming oxygen. We could hypothesize that once the oxygen concentration reaches a value around the hypoxic threshold, the cells experience oxidative stress and produce VEGF that will later promote the migration of endothelial cells and neovascularization. This will in turn help perfuse the construct with oxygen and nutrients, supporting both the therapeutic cell population and the subsequent neurite outgrowth. Therefore, if we optimize the construct by identifying the design that yields the maximal viable cell density and most favorable VEGF gradients, we could potentially accelerate nerve regeneration. Moreover, if in the future a more comprehensive database of cell and material‐type specific parameters is collated by repeating the in vitro experiments using different cell types, the mathematical model can be extended, allowing researchers to compare the behavior of different therapeutic cells under the same NRC configurations.

## CONFLICTS OF INTEREST

The authors declare no conflicts of interest.

## Data Availability

The data that support the findings of this study are openly available.

## References

[bit28105-bib-0001] Ara, J. , Fekete, S. , Zhu, A. , & Frank, M. (2010). Characterization of neural stem/progenitor cells expressing VEGF and its receptors in the subventricular zone of newborn piglet brain. Neurochemical Research, 35(9), 1455–1470. 10.1007/s11064-010-0207-2 20552272

[bit28105-bib-0002] Ardakani, A. G. , Cheema, U. , Brown, R. A. , & Shipley, R. J. (2014). Quantifying the correlation between spatially defined oxygen gradients and cell fate in an engineered three‐dimensional culture model. Journal of the Royal Society, Interface, 11(98), 20140501. 10.1098/rsif.2014.0501 24966240 PMC4233699

[bit28105-bib-0003] Aubert, A. , & Costalat, R. (2005). Interaction between astrocytes and neurons studied using a mathematical model of compartmentalized energy metabolism. Journal of Cerebral Blood Flow & Metabolism, 25(11), 1476–1490. 10.1038/sj.jcbfm.9600144 15931164

[bit28105-bib-0004] Barros, L. F. , Bittner, C. X. , Loaiza, A. , & Porras, O. H. (2007). A quantitative overview of glucose dynamics in the gliovascular unit. GLIA, 55(12), 1222–1237. 17659523 10.1002/glia.20375

[bit28105-bib-0005] Brown, R. A. , Wiseman, M. , Chuo, C.‐B. , Cheema, U. , & Nazhat, S. N. (2005). Ultrarapid engineering of biomimetic materials and tissues: Fabrication of nano‐ and microstructures by plastic compression. Advanced Functional Materials, 15(11), 1762–1770. 10.1002/adfm.200500042

[bit28105-bib-0006] Cain, J. T. , Berosik, M. A. , Snyder, S. D. , Crawford, N. F. , Nour, S. I. , Schaubhut, G. J. , & Darland, D. C. (2014). Shifts in the vascular endothelial growth factor isoforms result in transcriptome changes correlated with early neural stem cell proliferation and differentiation in mouse forebrain. Developmental Neurobiology, 74(1), 63–81. 10.1002/dneu.22130 24124161 PMC4096862

[bit28105-bib-0007] Campolongo, F. , Cariboni, J. , & Saltelli, A. (2007). An effective screening design for sensitivity analysis of large models. Environmental Modelling & Software, 22(10), 1509–1518. 10.1016/j.envsoft.2006.10.004

[bit28105-bib-0008] Carriel, V. , Alaminos, M. , Garzón, I. , Campos, A. , & Cornelissen, M. (2014). Tissue engineering of the peripheral nervous system. Expert Review of Neurotherapeutics, 14(3), 301–318. 10.1586/14737175.2014.887444 24506662

[bit28105-bib-0009] Carrier, R. L. , Rupnick, M. , Langer, R. , Schoen, F. J. , Freed, L. E. , & Vunjak‐Novakovic, G. (2002). Perfusion improves tissue architecture of engineered cardiac muscle. Tissue Engineering, 8(2), 175–188. 10.1089/107632702753724950 12031108

[bit28105-bib-0010] Cattin, A.‐L. , Burden, J. J. , Van Emmenis, L. , Mackenzie, F. E. , Hoving, J. J. , Garcia Calavia, N. , Guo, Y. , Rosenberg, L. H. , Quereda, V. , Jamecna, D. , Napoli, I. , Parrinello, S. , Enver, T. , Ruhrberg, C. , & Lloyd, A. C. (2015). Macrophage‐induced blood vessels guide schwann cell‐mediated regeneration of peripheral nerves. Cell, 162(5), 1127–1139. 10.1016/j.cell.2015.07.021 26279190 PMC4553238

[bit28105-bib-0011] Cheema, U. , Brown, R. A. , Alp, B. , & MacRobert, A. J. (2007). Spatially defined oxygen gradients and vascular endothelial growth factor expression in an engineered 3D cell model. Cellular and Molecular Life Sciences, 65(1), 177–186. 10.1007/s00018-007-7356-8 PMC11131842 17994289

[bit28105-bib-0012] Cheema, U. , Rong, Z. , Kirresh, O. , MacRobert, A. J. , Vadgama, P. , & Brown, R. A. (2012). Oxygen diffusion through collagen scaffolds at defined densities: Implications for cell survival in tissue models. Journal of Tissue Engineering and Regenerative Medicine, 6(1), 77–84. 10.1002/term.402 21312340

[bit28105-bib-0013] Chen, R. R. , Silva, E. A. , Yuen, W. W. , & Mooney, D. J. (2007). Spatio–temporal VEGF and PDGF delivery patterns blood vessel formation and maturation. Pharmaceutical Research, 24(2), 258–264. 10.1007/s11095-006-9173-4 17191092

[bit28105-bib-0014] Chung, C. A. , Yang, C. W. , & Chen, C. W. (2006). Analysis of cell growth and diffusion in a scaffold for cartilage tissue engineering. Biotechnology and Bioengineering, 94(6), 1138–1146. 10.1002/bit.20944 16586509

[bit28105-bib-0015] Cochran, D. M. , Fukumura, D. , Ancukiewicz, M. , Carmeliet, P. , & Jain, R. K. (2006). Evolution of oxygen and glucose concentration profiles in a tissue‐mimetic culture system of embryonic stem cells. Annals of Biomedical Engineering, 34(8), 1247–1258. 10.1007/s10439-006-9134-4 16832606

[bit28105-bib-0016] Coy, R. , Al‐Badri, G. , Kayal, C. , O'Rourke, C. , Kingham, P. J. , Phillips, J. B. , & Shipley, R. J. (2020). Combining in silico and in vitro models to inform cell seeding strategies in tissue engineering. Journal of the Royal Society Interface, 17(164), 20190801. 10.1098/rsif.2019.0801 32208821 PMC7115239

[bit28105-bib-0017] Coy, R. , Berg, M. , Phillips, J. B. , & Shipley, R. (2021). Modelling‐informed cell‐seeded nerve repair construct designs for treating peripheral nerve injuries. PLoS Computational Biology, 17(7), e1009142. Retrieved from. 10.1371/journal.pcbi.1009142 34237052 PMC8266098

[bit28105-bib-0018] Coy, R. H. , Evans, O. R. , Phillips, J. B. , & Shipley, R. J. (2018). An integrated theoretical‐experimental approach to accelerate translational tissue engineering. Journal of Tissue Engineering and Regenerative Medicine, 12(1), e53–e59. 10.1002/term.2346 27792286 PMC5811925

[bit28105-bib-0019] Dienel, G. A. , Cruz, N. F. , Sokoloff, L. , & Driscoll, B. F. (2017). Determination of glucose utilization rates in cultured astrocytes and neurons with [14C]deoxyglucose: Progress, pitfalls, and discovery of intracellular glucose compartmentation. Neurochemical Research, 42(1), 50–63. 10.1007/s11064-015-1650-x 26141225

[bit28105-bib-0020] Donzelli, R. , Capone, C. , Sgulò, F. G. , Mariniello, G. , & Maiuri, F. (2016). Vascularized nerve grafts: An experimental study. Neurological Research, 38(8), 669–677. 10.1080/01616412.2016.1198527 27349271

[bit28105-bib-0021] Duarte, J. M. N. , Morgenthaler, F. D. , Lei, H. , Poitry‐Yamate, C. , & Gruetter, R. (2009). Steady‐state brain glucose transport kinetics re‐evaluated with a four‐state conformational model. Frontiers in Neuroenergetics, 1, 6. 10.3389/neuro.14.006.2009 20027232 PMC2795468

[bit28105-bib-0023] Fong, G.‐H. (2008). Mechanisms of adaptive angiogenesis to tissue hypoxia. Angiogenesis, 11(2), 121–140. 10.1007/s10456-008-9107-3 18327686

[bit28105-bib-0024] Mac Gabhann, F. , Ji, J. W. , & Popel, A. S. (2007). VEGF gradients, receptor activation, and sprout guidance in resting and exercising skeletal muscle. Journal of Applied Physiology, 102(2), 722–734. 17038488 10.1152/japplphysiol.00800.2006

[bit28105-bib-0025] Mac Gabhann, F. , Yang, M. T. , & Popel, A. S. (2005). Monte Carlo simulations of VEGF binding to cell surface receptors in vitro. Biochimica et Biophysica Acta (BBA)—Molecular Cell Research, 1746(2), 95–107. 10.1016/j.bbamcr.2005.09.004 16257459

[bit28105-bib-0026] Garcia Sanchez, D. , Lacarrière, B. , Musy, M. , & Bourges, B. (2014). Application of sensitivity analysis in building energy simulations: Combining first‐ and second‐order elementary effects methods. Energy and Buildings, 68, 741–750. 10.1016/j.enbuild.2012.08.048

[bit28105-bib-0027] Georgiou, M. , Golding, J. P. , Loughlin, A. J. , Kingham, P. J. , & Phillips, J. B. (2015). Engineered neural tissue with aligned, differentiated adipose‐derived stem cells promotes peripheral nerve regeneration across a critical sized defect in rat sciatic nerve. Biomaterials, 37, 242–251. 10.1016/j.biomaterials.2014.10.009 25453954

[bit28105-bib-0028] Grinsell, D. , & Keating, C. P. (2014). Peripheral nerve reconstruction after injury: A review of clinical and experimental therapies. BioMed Research International, 2014, 698256. 10.1155/2014/698256 25276813 PMC4167952

[bit28105-bib-0029] Gu, W. , Gaeta, X. , Sahakyan, A. , Chan, A. B. , Hong, C. S. , Kim, R. , Braas, D. , Lowry, W. E. , & Christofk, H. R. (2016). Glycolytic metabolism plays a functional role in regulating human pluripotent stem cell state. Cell Stem Cell, 19(4), 476–490. 27618217 10.1016/j.stem.2016.08.008 PMC5055460

[bit28105-bib-0030] Han, P. , & Bartels, D. M. (1996). Temperature dependence of oxygen diffusion in H_2_O and D_2_O. The Journal of Physical Chemistry, 100(13), 5597–5602. 10.1021/jp952903y

[bit28105-bib-0031] Haselgrove, J. C. , Shapiro, I. M. , & Silverton, S. F. (1993). Computer modeling of the oxygen supply and demand of cells of the avian growth cartilage. American Journal of Physiology‐Cell Physiology, 265(2), C497–C506. 10.1152/ajpcell.1993.265.2.C497 8368277

[bit28105-bib-0032] Hashimoto, T. , & Shibasaki, F. (2015). Hypoxia‐inducible factor as an angiogenic master switch. Frontiers in Pediatrics, 3, 33. https://www.frontiersin.org/article/10.3389/fped.2015.00033 25964891 10.3389/fped.2015.00033 PMC4408850

[bit28105-bib-0033] Herculano‐Houzel, S. (2011). Scaling of brain metabolism with a fixed energy budget per neuron: implications for neuronal activity, plasticity and evolution. PLoS One, 6(3), e17514. 10.1371/journal.pone.0017514 21390261 PMC3046985

[bit28105-bib-0034] Herman, J. , & Usher, W. (2017). SALib: An open‐source Python library for sensitivity analysis. Journal of Open Source Software, 2(9), 97.

[bit28105-bib-0035] Hobson, M. I. , Green, C. J. , & TerenghiI, G. (2000). VEGF enhances intraneural angiogenesis and improves nerve regeneration after axotomy. Journal of Anatomy, 197(4), 591–605. 10.1046/j.1469-7580.2000.19740591.x 11197533 PMC1468175

[bit28105-bib-0036] Hsu, S. H. , Kuo, W. C. , Chen, Y. T. , Yen, C. T. , Chen, Y. F. , Chen, K. S. , & Huang, W. C. (2013). New nerve regeneration strategy combining laminin‐coated chitosan conduits and stem cell therapy. Acta Biomaterialia, 9(5), 6606–6615. 10.1016/j.actbio.2013.01.025 23376237

[bit28105-bib-0037] Huang, H.‐M. , Ismail‐Beigi, F. , & Muzic, R. F., Jr. (2011). A new Michaelis‐Menten‐based kinetic model for transport and phosphorylation of glucose and its analogs in skeletal muscle. Medical Physics, 38(8), 4587–4599. 10.1118/1.3599034 21928632 PMC3173937

[bit28105-bib-0038] Jiang M. , Luo Y. P. , & Yang S. Y. (2007). Stochastic convergence analysis and parameter selection of the standard particle swarm optimization algorithm. Information Processing Letters 102(1), 816. 10.1016/j.ipl.2006.10.005

[bit28105-bib-0039] Jin, K. , Mao, X. O. , Batteur, S. P. , McEachron, E. , Leahy, A. , & Greenberg, D. A. (2001). Caspase‐3 and the regulation of hypoxic neuronal death by vascular endothelial growth factor. Neuroscience, 108(2), 351–358. 10.1016/S0306-4522(01)00154-3 11734367

[bit28105-bib-0040] Kalladka, D. , Sinden, J. , Pollock, K. , Haig, C. , McLean, J. , Smith, W. , Mcconnachie, A. , Bath, P. M. , Dunn, L. , & Muir, K. W. (2016). Human neural stem cells in patients with chronic ischaemic stroke (PISCES): A phase 1, first‐in‐man study. The Lancet, 388(10046), 787–796. 10.1016/S0140-6736(16)30513-X 27497862

[bit28105-bib-0041] Kennedy, J. , & Eberhart, R. (1995). Particle swarm optimization. In *Proceedings of ICNN'95‐International Conference on Neural Networks*. IEEE (Vol. 4, pp. 1942–1948).

[bit28105-bib-0042] Köhn‐Luque, A. , De Back, W. , Yamaguchi, Y. , Yoshimura, K. , Herrero, M. A. , & Miura, T. (2013). Dynamics of VEGF matrix‐retention in vascular network patterning. Physical Biology, 10(6), 66007. 10.1088/1478-3975/10/6/066007 24305433

[bit28105-bib-0043] Lad, S. P. , Nathan, J. K. , Schubert, R. D. , & Boakye, M. (2010). Trends in median, ulnar, radial, and brachioplexus nerve injuries in the United States. Neurosurgery, 66(5), 953–960. 10.1227/01.NEU.0000368545.83463.91 20414978

[bit28105-bib-0044] Lee, H.‐L. , Lee, H. Y. , Yun, Y. , Oh, J. , Che, L. , Lee, M. , & Ha, Y. (2016). Hypoxia‐specific, VEGF‐expressing neural stem cell therapy for safe and effective treatment of neuropathic pain. Journal of Controlled Release, 226, 21–34. 10.1016/j.jconrel.2016.01.047 26826306

[bit28105-bib-0045] Lewis, M. C. , MacArthur, B. D. , Malda, J. , Pettet, G. , & Please, C. P. (2005). Heterogeneous proliferation within engineered cartilaginous tissue: The role of oxygen tension. Biotechnology and Bioengineering, 91(5), 607–615. 10.1002/bit.20508 16025534

[bit28105-bib-0046] Lim, T. K. Y. , Shi, X. Q. , Johnson, J. M. , Rone, M. B. , Antel, J. P. , David, S. , & Zhang, J. (2015). Peripheral nerve injury induces persistent vascular dysfunction and endoneurial hypoxia, contributing to the genesis of neuropathic pain. The Journal of Neuroscience, 35(8), 3346 LP–3343359. 10.1523/JNEUROSCI.4040-14.2015 25716835 PMC6605560

[bit28105-bib-0047] Magliaro, C. , Mattei, G. , Iacoangeli, F. , Corti, A. , Piemonte, V. , & Ahluwalia, A. (2019). Oxygen consumption characteristics in 3D constructs depend on cell density. Frontiers in Bioengineering and Biotechnology, 7, 251. https://www.frontiersin.org/article/10.3389/fbioe.2019.00251 31649925 10.3389/fbioe.2019.00251 PMC6796794

[bit28105-bib-0048] McMurtrey, R. J. (2015). Analytic models of oxygen and nutrient diffusion, metabolism dynamics, and architecture optimization in three‐dimensional tissue constructs with applications and insights in cerebral organoids. Tissue Engineering Part C: Methods, 22(3), 221–249. 10.1089/ten.tec.2015.0375 PMC5029285 26650970

[bit28105-bib-0049] Miranda, L. J. (2018). PySwarms: A research toolkit for Particle Swarm Optimization in Python. Journal of Open Source Software, 3(21), 433.

[bit28105-bib-0050] Morris, M. D. (1991). Factorial sampling plans for preliminary computational experiments. Technometrics, 33(2), 161–174.

[bit28105-bib-0051] Newrick, P. G. , Wilson, A. J. , Jakubowski, J. , Boulton, A. J. , & Ward, J. D. (1986). Sural nerve oxygen tension in diabetes. British Medical Journal (Clinical Research Ed.), 293(6554), 1053–1054. http://www.ncbi.nlm.nih.gov/pmc/articles/PMC1341910/ 3094772 10.1136/bmj.293.6554.1053 PMC1341910

[bit28105-bib-0053] O'rourke, C. , Day, A. , Murray‐Dunning, C. , Thanabalasundaram, L. , Cowan, J. , Stevanato, L. , Grace, N. , Drake, R. , Sinden, J. , & Phillips, J. B. (2018). An allogeneic ‘off the shelf’ therapeutic strategy for peripheral nerve tissue engineering using clinical grade human neural stem cells. Scientific Reports, 8, 2951. 10.1038/s41598-018-20927-8 29440680 PMC5811594

[bit28105-bib-0054] Panagopoulos, G. N. , Megaloikonomos, P. D. , & Mavrogenis, A. F. (2017). The present and future for peripheral nerve regeneration. Orthopedics, 40(1), e141–e156. 27783836 10.3928/01477447-20161019-01

[bit28105-bib-0055] Patzer, J. F. II . (2004). Oxygen consumption in a hollow fiber bioartificial liver—revisited. Artificial Organs, 28(1), 83–98. 10.1111/j.1525-1594.2004.07150.x 14720293

[bit28105-bib-0056] Radisic, M. , Malda, J. , Epping, E. , Geng, W. , Langer, R. , & Vunjak‐Novakovic, G. (2006). Oxygen gradients correlate with cell density and cell viability in engineered cardiac tissue. Biotechnology and Bioengineering, 93(2), 332–343. 10.1002/bit.20722 16270298

[bit28105-bib-0057] Rayner, M. L. D. , Day, A. G. E. , Bhangra, K. S. , Sinden, J. , & Phillips, J. B. (2021). Engineered neural tissue made using clinical‐grade human neural stem cells supports regeneration in a long gap peripheral nerve injury model. Acta Biomaterialia, 135, 203–213. 10.1016/j.actbio.2021.08.030 34455110

[bit28105-bib-0058] Rouwkema, J. , Rivron, N. C. , & van Blitterswijk, C. A. (2008). Vascularization in tissue engineering. Trends in Biotechnology, 26(8), 434–441. 10.1016/j.tibtech.2008.04.009 18585808

[bit28105-bib-0059] Rutkowski, G. E. , & Heath, C. A. (2002). Development of a bioartificial nerve graft. i. design based on a reaction–diffusion model. Biotechnology Progress, 18(2), 362–372. 10.1021/bp020300f 11934308

[bit28105-bib-0060] Saltelli, A. , Tarantola, S. , Campolongo, F. , & Ratto, M. (2004). Sensitivity analysis in practice: A guide to assessing scientific models (Vol. 1). Wiley Online Library.

[bit28105-bib-0061] Schuh, C. M. A. P. , Day, A. G. E. , Redl, H. , & Phillips, J. (2018). An Optimized Collagen‐Fibrin Blend Engineered Neural Tissue Promotes Peripheral Nerve Repair. Tissue Engineering—Part A, 24(17–18), 1332–1340. 10.1089/ten.tea.2017.0457 29652609 PMC6150938

[bit28105-bib-0062] Shipley, R. J. , Jones, G. W. , Dyson, R. J. , Sengers, B. G. , Bailey, C. L. , Catt, C. J. , & Please, C. P. (2009). Design criteria for a printed tissue engineering construct: A mathematical homogenization approach. Journal of Theoretical Biology, 259(3), 489–502. 10.1016/j.jtbi.2009.03.037 19361531

[bit28105-bib-0063] Sielaff, T. D. , Nyberg, S. L. , Rollins, M. D. , Hu, M. Y. , Amiot, B. , Lee, A. , & Cerra, F. B. (1997). Characterization of the three‐compartment gel‐entrapment porcine hepatocyte bioartificial liver. Cell Biology and Toxicology, 13(4), 357–364. 10.1023/A:1007499727772 9298256

[bit28105-bib-0064] Smith, E. J. , Stroemer, R. P. , Gorenkova, N. , Nakajima, M. , Crum, W. R. , Tang, E. , Stevanato, L. , & Modo, M. (2012). Implantation site and lesion topology determine efficacy of a human neural stem cell line in a rat model of chronic stroke. Stem Cells, 30(4), 785–796. 10.1002/stem.1024 22213183

[bit28105-bib-0065] Stevanato, L. , Corteling, R. L. , Stroemer, P. , Hope, A. , Heward, J. , Miljan, E. A. , & Sinden, J. D. (2009). c‐MycERTAM transgene silencing in a genetically modified human neural stem cell line implanted into MCAo rodent brain. BMC Neuroscience, 10, 86. 10.1186/1471-2202-10-86 19622162 PMC2725042

[bit28105-bib-0066] Streeter, I. , & Cheema, U. (2011). Oxygen consumption rate of cells in 3D culture: The use of experiment and simulation to measure kinetic parameters and optimise culture conditions. Analyst, 136(19), 4013–4019. 10.1039/C1AN15249A 21804989

[bit28105-bib-0067] Suhaimi, H. , Wang, S. , & Das, D. B. (2015). Glucose diffusivity in cell culture medium. Chemical Engineering Journal, 269, 323–327. 10.1016/j.cej.2015.01.130

[bit28105-bib-0068] Teslaa, T. , & Teitell, M. A. (2015). Pluripotent stem cell energy metabolism: An update. The EMBO Journal, 34(2), 138–153. 10.15252/embj.201490446 25476451 PMC4337063

[bit28105-bib-0069] Tuck, R. R. , Schmelzer, J. D. , & Low, P. A. (1984). Endoneurial blood flow and oxygen tension in the sciatic nerves of rats with experimental diabetic neuropathy. Brain: A Journal of Neurology, 107(3), 935–950. 10.1093/brain/107.3.935 *Pt 3*. 6478183

[bit28105-bib-0070] Vempati, P. , Popel, A. S. , & Mac Gabhann, F. (2011). Formation of VEGF isoform‐specific spatial distributions governing angiogenesis: Computational analysis. BMC Systems Biology, 5, 59. 10.1186/1752-0509-5-59 21535871 PMC3113235

[bit28105-bib-0071] Vempati, P. , Popel, A. S. , & Mac Gabhann, F. (2014). Extracellular regulation of VEGF: Isoforms, proteolysis, and vascular patterning. Cytokine & Growth Factor Reviews, 25(1), 1–19. 10.1016/j.cytogfr.2013.11.002 24332926 PMC3977708

[bit28105-bib-0072] Wagner, B. A. , Venkataraman, S. , & Buettner, G. R. (2011). The rate of oxygen utilization by cells. Free Radical Biology & Medicine, 51(3), 700–712. 10.1016/j.freeradbiomed.2011.05.024 21664270 PMC3147247

[bit28105-bib-0073] Wang, L. , Shi, Q. , Cai, Y. , Chen, Q. , Guo, X. , & Li, Z. (2020). Mechanical–chemical coupled modeling of bone regeneration within a biodegradable polymer scaffold loaded with VEGF. Biomechanics and Modeling in Mechanobiology, 19(6), 2285–2306. 10.1007/s10237-020-01339-y 32488347

[bit28105-bib-0074] Wu, D.‐Q. , Zhang, G.‐L. , Shen, C. , Zhao, Q. , Li, H. , & Meng, Q. (2005). Evaluation of diffusion in gel entrapment cell culture within hollow fibers. World Journal of Gastroenterology, 11(11), 1599–1604. 10.3748/wjg.v11.i11.1599 15786534 PMC4305938

[bit28105-bib-0075] Yang, M. , Rawson, J. L. , Zhang, E. W. , Arnold, P. B. , Lineaweaver, W. , & Zhang, F. (2011). Comparisons of outcomes from repair of median nerve and ulnar nerve defect with nerve graft and tubulization: A meta‐analysis. Journal of Reconstructive Microsurgery, 27(08), 451–460. 21796581 10.1055/s-0031-1281526

[bit28105-bib-0076] Zhong, Y. , Motavalli, M. , Wang, K.‐C. , Caplan, A. I. , Welter, J. F. , & Baskaran, H. (2018). Dynamics of intrinsic glucose uptake kinetics in human mesenchymal stem cells during chondrogenesis. Annals of Biomedical Engineering, 46(11), 1896–1910. 10.1007/s10439-018-2067-x 29948374 PMC6204100

[bit28105-bib-0077] Van Zijl, P. C. M. , Davis, D. , Eleff, S. M. , Moonen, C. T. W. , Parker, R. J. , & Strong, J. M. (1997). Determination of cerebral glucose transport and metabolic kinetics by dynamic MR spectroscopy. American Journal of Physiology‐Endocrinology and Metabolism, 273(6), E1216–E1227. 10.1152/ajpendo.1997.273.6.E1216 9435539

[bit28105-bib-0078] Zochodne, D. W. , Ho, L. T. , & Allison, J. A. (1994). Dorsal root ganglia microenvironment of female BB Wistar diabetic rats with mild neuropathy. Journal of the Neurological Sciences, 127(1), 36–42. 10.1016/0022-510X(94)90132-5 7699389

